# Arteriovenous fistula creation by nephrologist and its outcomes: a prospective cohort study from Vietnam

**DOI:** 10.1186/s12882-023-03123-3

**Published:** 2023-04-04

**Authors:** Bach Nguyen, Minh Cuong Duong, Huynh Ngoc Diem Tran, Kim Que Do, Kim Thai Thien Nguyen

**Affiliations:** 1 Department of Nephrology and Dialysis, Thong Nhat Hospital, 1 Ly Thuong Kiet Street, Ward 7, Tân Binh District, Ho Chi Minh City, Vietnam; 2 School of Population Health, University of New South Wales, Sydney, NSW Australia; 3 Department of Cardiovascular Surgery, Thong Nhat Hospital, 1 Ly Thuong Kiet Street, Ward 7, Tân Binh District, Ho Chi Minh City, Vietnam; 4 Faculty of Pharmacy, University of Medicine and Pharmacy at Ho Chi Minh City, 43 Dinh Tien Hoang Street, Ben Nghe Ward, District 1, Ho Chi Minh City, Vietnam

**Keywords:** Hemodialysis, Arterial venous fistulas, Anastomosis, AVF creation, Nephrologist

## Abstract

**Background:**

Arteriovenous fistula (AVF) is the gold standard vascular access for effective hemodialysis. There is a growing interest in AVF creations performed by nephrologists to help reduce vascular surgeons’ workload and enhance the timely treatment of patients with end-stage renal disease (ESRD). However, little is known about the feasibility and effectiveness of this approach in the low-resource settings. We examined the AVF surgical success and failure rates and associated predictors as well as early complications of AVF creations by a trained nephrologist with supports from vascular surgeons in Vietnam.

**Methods:**

A prospective cohort study was conducted on all adult ESRD patients at the Hemodialysis Department of Thong Nhat Hospital between April 2018 and October 2020. Information on demographic characteristics, comorbidities, and AVF creations was collected using a standardized questionnaire. All patients were followed up until 18 weeks post-surgery.

**Results:**

Among 100 patients with a mean age of 61.22 ± 17.11 years old, male accounted for 54%. Common causes of ESRD included hypertension (57%) and diabetes (32%). Just more than half (52%) of them reported having an AVF creation prior to ESRD. The successful first-time AVF creation rate was 98% (13/99, 95%CI: 8.74–21.18%). The primary and secondary AVF failure rates were 13.13% (13/99, 95%CI: 8.74–21.18%) and 16.87% (14/83, 95%CI: 10.32–26.25%), respectively. Early complications included bleeding (1%) and early thrombosis of the anastomosis (2%). There was a statistically significant association between age and primary AVF failure (P = 0.005) and between operation time and secondary AVF failure (P = 0.038).

**Conclusions:**

AVF creations performed by well-trained and skilled interventional nephrologists with supports from vascular surgeons can result in favorable short- and long-term outcomes. It is important to follow up older patients and those with a long operation time to detect AVF failures. A standardized AVF creation training program and practice for nephrologists is needed to increase successful rates.

## Background

There has been an increased number of patients with end-stage renal disease (ESRD) worldwide including Vietnam that leads to the high demand for hemodialysis [1, 2]. Arteriovenous fistulas (AVF) are the gold standard vascular access for chronic hemodialysis treatment because of lower risks of complications associated with long-term use compared to arteriovenous grafts (AVG) and permanent cuff catheters [3, 4]. Early referral of patients with ESRD to vascular surgeons is crucial to allow adequate time for planning an AVF creation and enable it to mature [2].

In Vietnam, ESRD patients requiring chronic hemodialysis treatment do usually not have mature and functioning AVFs in place for the timely start of hemodialysis due to gaps in the management process of earlier stages of chronic kidney disease (CKD) [5, 6]. The management of vascular access in patients with CKD has not been standardized and mainly depends on the availability of human resources. In detail, in small hospitals and satellite dialysis centers, CKD patients are monitored for vascular access mainly by dialysis nurses due to shortages of both nephrologists and vascular surgeons [5, 7–9]. In large hospitals, nephrologists and dialysis nurses are responsible for this process, while vascular surgeon consultations are only available for selected, difficult cases [5]. Therefore, like other developing countries AVF creations prior to initiation of hemodialysis are still rare in Vietnam [5]. Most CKD patients require an urgent start of hemodialysis with a temporary central venous catheter which may lead to infections and other catheter-associated complications [5, 6]. Therefore, there is an urgent need for timely AVF creations for these patients in such situations [10, 11]. In this context, there is a growing interest in AVF creations performed by nephrologists to help reduce surgeons’ workload and enhance the effective management of ESRD patients [2, 12, 13]. Despite this, little is known about the feasibility and effectiveness of surgically created AVF by nephrologists not only in Vietnam but also globally [7]. This study was conducted to examine the AVF surgical success and failure rates as well as early complications of AVF creations performed by a trained nephrologist with supports from vascular surgeons in Vietnam. We also aimed to identify predictors of AVF failures.

## Method

### Context and design of study

A prospective cohort study was conducted at the Hemodialysis Department of Thong Nhat Hospital (TNH) between April 2018 and October 2020. TNH is a 1,500-bed, tertiary teaching, geriatric hospital in southern Vietnam [14]. The Hemodialysis Department receives CKD patients from across southern Vietnam and provides an average of 1,680 dialysis sessions monthly. The study was approved by the TNH’s Ethics Committee (reference No. TN-13-07-2018).

All patients included in this study met the following criteria: (1) being 18 years or older, (2) being diagnosed with ESRD and requiring an AVF for chronic hemodialysis, (3) having a good venous and arterial anatomy assessed by qualified physicians (the radial and ulnar pulses are good; the Allen test is negative; the cephalic vein of the forearm is straight, within 1 cm from the surface of the forearm and has no clinical evidence of obstruction; and/or minimum venous and arterial diameter of more than 2 mm on Doppler ultrasound), (4) having no evidence of severe heart failure (ejection fraction (EF) > 35%), and (5) being able to complete the study. Those who did not meet the inclusion criteria were consulted by a team of qualified nephrologists, vascular surgeons and cardiologists and were advised for undertaking continuous ambulatory peritoneal dialysis (CAPD) or hemodialysis using permanent catheter. Given that patients’ edematous limbs may induce difficulties during the AVF creation, patients could only undertake the AVF creation when resolution of edema was achieved [15].

A standardized questionnaire was used to collect study participants’ baseline information at the time of AVF creation including demographic characteristics (age and gender), etiology of ESRD, blood pressure, hemoglobin and serum albumin levels, dyslipidemia (serum cholesterol > 5,2 mmol/L, triglyceride > 1,7 mmol/L, LDL-cholesterol > 2,58 mmol/L, and/or HDL-cholesterol < 1,03nmmol/L), preoperative vascular mapping with Doppler ultrasound examination of both arms for those with a poor vasculature identified by clinical examination. Information on the AVF creation was also recorded and included history of AVF creation, frequency of AVF creation procedures, types of arteriovenous anastomosis (end-to-side (ETS) or side-to-side (STS) technique), length of anastomosis, operation time (the duration from skin incision to stitched skin), early complications of the AVF creation (bleeding and thrombosis of the anastomosis), and primary and secondary AVF failure rates.

### AVF creation procedure

Patients with generalized edema were well managed prior to the operation. All surgeries were performed in the operation theater of TNH. A nephrologist performed the operation under supervision of a qualified vascular surgeon. The nephrologist had completed a 6-month training on AVF creation and been an assistant surgeon of 60 successful AVF creations before the presenting study was conducted. The creation of AVF was performed under local anesthesia with 2% lidocaine and using running sutures with 7 − 0 Prolene. A longitudinal 3–4 cm skin incision was used provided that it was found to give a good access to both vein and artery [16]. The ETS technique was utilized to create AVF due to its superior results compared to the STS approach [17]. However, the latter technique was also used and based on the nephrologist’s clinical judgement [18]. Patients who were not successful with the first AVF creation were arranged to undertake a second procedure within 24 h by the same operation team. Intraoperative heparin was used to prevent clotting.

### Definitions of follow-up, successful first-time AVF creation, and primary and secondary AVF failures

All patients were followed up until 18 weeks post-surgery. A maturated AVF has been confirmed by experienced nephrologists’ examination and ultrasound. A maturated AVF is defined as a soft and easily compressible vein with a continuous audible bruit (i.e., an audible low pitched continuous systolic and diastolic bruit) and a palpable thrill near the anastomosis extending along the vein for a varying distance [19, 20]. The AVF also has an adequate length and is superficial enough to be punctured [19, 20]. Regarding ultrasound, a maturated AVF is defined as the vein diameter measurement of at least 4 mm with a blood flow rate of at least 500 ml/minute [20–22]. Therefore, a successful first-time AVF creation was defined as the presence of AVF blood flow which can be confirmed by physical examination of both the nephrologist and vascular surgeon involved in the operation during 24 h after the first operation (a detection of a palpable thrill and continuous bruit was considered as an indicator for successful AVF creation). Primary AVF failure was defined as a permanent failure of the newly created AVF before it became suitable for hemodialysis treatment characterized as an inadequate maturation, thrombosis, failure of the first and subsequent cannulations, and other complications leading to nonfunctional AVFs within a 6-week period after the surgery [17]. Secondary AVF failure was defined as a permanent failure of the newly created AVF after it had been used for hemodialysis for 18 weeks [17]. Indeed, the standard definitions of early and late dialysis suitability failures require a close follow-up of patients three and six months, respectively [20] which cannot be carried out in Vietnam due to the lack of human resources and an effective preparation program for CKD patients. Therefore, our definitions of primary and late AVF failures were revised accordingly to meet the Vietnam context. In addition, our AVF failure definitions were exclusively based on the clinical examination by the interventional nephrologist and vascular surgeon. Ultrasound was only utilized in case of suspected inadequate AVF maturation or thrombosis to address the shortage of vascular ultrasound specialists.

### Statistical analysis

Data were managed and analyzed using the Statistical Package for the Social Sciences (SPSS) version 26 (IBM). A descriptive analysis of the study population’s baseline characteristics was carried out. Continuous variables were displayed as mean ± one standard deviation (SD), while categorical variables were presented as an absolute count and percentage (%). The proportions of successful AVF creations and AVF failures as well as associated 95% confidence intervals (95%CIs) were calculated for comparison purposes. Chi-squared test was used to compare categorical data, while student’s t-test was used to compare continuous data. Alpha was set at 5% level.

## Results

### Baseline characteristics of study participants

**Fig. 1:**
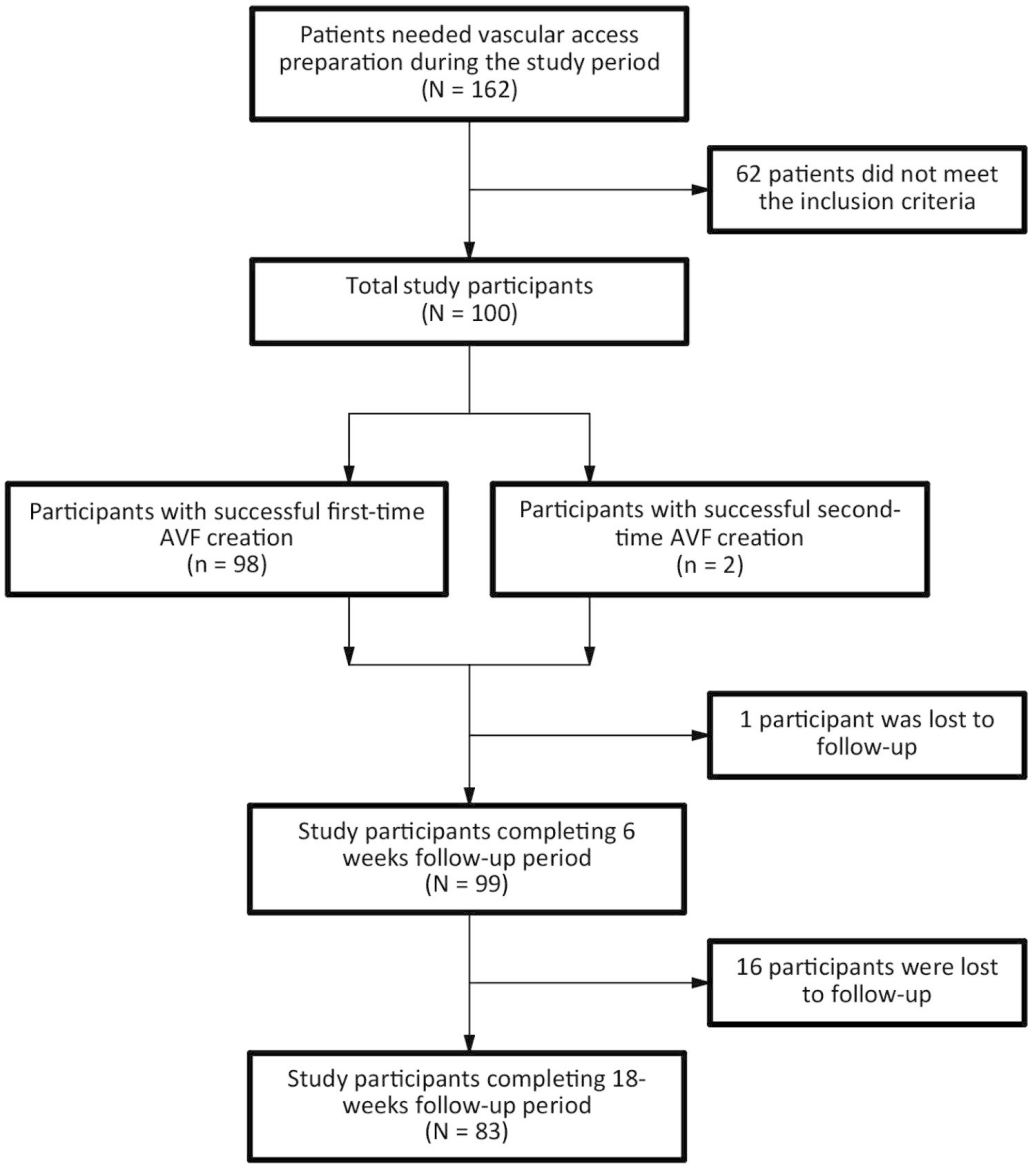
Flowchart of study participants

There was a total of 162 ESRD patients who needed a vascular access preparation during the study period (Fig. 1). Of these patients, 62 (38%) did not meet the inclusion criteria and were arranged to undertake other types of vascular access. The remaining 100 patients with a mean age of 61.22 ± 17.11 years old and a mean systolic blood pressure of 136.80 ± 16.38 mmHg were entered into the study (Table 1). Of these 100 participants, male and dyslipidemia accounted for 54% and 34%, respectively. The mean hemoglobin and serum albumin levels were 9.06 ± 1.37 g/dL and 32.66 ± 5.73 g/dL, respectively, while the mean ejection fraction was 66.11 ± 10.74%. The most common causes of ESRD included hypertension (57%) followed by diabetes mellitus (32%). More than one third (37%) of participants used a temporary catheter for an urgent hemodialysis, while 11% had been on chronic hemodialysis using a permanent catheter. Less than one third (29%) of participants received a preoperative vascular mapping with Doppler ultrasound. Just more than half (52%) of participants undertook AVF creations before hemodialysis initiation.

**Table 1:** Baseline characteristics of 100 study participants

Characteristics	Summary statistics* (N = 100)
Demographics	
Age (years)	61.22 ± 17.11
Age $\ge$ 60	62 (62)
Male	54 (54)
Etiologies of end stage renal disease Hypertension Diabetes mellitus Others**	57 (57) 32 (32) 11 (11)
Blood pressure (mmHg) Systolic Diastolic	136.80 ± 16.38 76.10 ± 8.40
Vascular access preparation	
AVF creation before hemodialysis initiation	52 (52)
Hemodialysis initiation using temporary central venous catheter Hemodialysis initiation using permanent catheter	37 (37) 11 (11)
Laboratory test results	
Hb (g/dL)	9.06 ± 1.37
Serum albumin (g/dL)	32.66 ± 5.73
Dyslipidemia	34
Ejection fraction (%)	66.11 ± 10.74
Availability of preoperative vascular mapping with Doppler ultrasound	29 (29)

*AVF: arteriovenous fistula, HB: hemoglobin*

*mean ± SD for continuous variables and n (%) for categorical variables

**Chronic glomerular diseases, polycystic kidney diseases, kidney stone, and unknown causes

### AVF creations and outcomes

Among 100 study participants, 94 underwent their first-time AVF creation at the time the study was conducted (Table 2). The mean operation time was 73.45 ± 21.03 min. ETS and STS techniques were used in 87 and 13 participants, respectively. Radiocephalic wrist AVF accounted for 80% of participants, and the associated mean length of anastomosis was 5.5 ± 0.5 mm. During 24 h after the operation, early complications including bleeding and early thrombosis of the anastomosis were recorded in 1 (1%) and 2 (2%) cases, respectively. Two participants required a second AVF procedure and thus, the successful first-time AVF creation rate was 98%. Among 99 participants completing the 6-week follow-up period, 13 had nonfunctional AVF. Thus, the primary AVF failure rate was 13.13% (13/99, 95%CI: 8.74–21.18%). Among 83 participants completing the 18-week follow-up period, 14 had nonfunctional AVF. Thus, the secondary AVF failure rate was 16.87% (14/83, 95%CI: 10.32–26.25%).

**Table 2:** Characteristics and outcomes of AVF creations among 100 study participants

Characteristics and outcomes of AVF creations	Summary statistics*
History of AVF creation First time Second time or more	94 (94) 6 (06)
Types of arteriovenous anastomosis End-to-side arteriovenous anastomosis Side-to-side arteriovenous anastomosis	87 (87) 13 (13)
Site of AVF creation The wrist (Radiocephalic fistula) The elbow (Brachiocephalic fistula and brachiobasilic fistula)	80 (80) 20 (20)
Length of anastomosis (mm) Anastomosis at the wrist Anastomosis at the elbow	5.5 ± 0.5 3.5 ± 0.5
Operation time (minutes)	73.45 ± 21.03
Early complications Bleeding at anastomosis Thrombosis of anastomosis	1 (1) 2 (2)

*mean ± SD for continuous variables and n (%) for categorical variables

### Risk factors for primary AVF failure

Age was significantly associated with primary AVF failure (P = 0.005) (Table 3). There were no significant differences between patients having primary AVF failures compared to those who did not with respect to sex, having diabetes and dyslipidemia, types of arteriovenous anastomosis, sites of AVF creation, operation time, and history of AVF creation (P > 0.05).

**Table 3:** Association between baseline and AVF creation characteristics and primary AVF failure among 100 participants

Characteristics	Primary AVF failure* (N = 99)		P value**	OR (95%CI)
	Yes (n = 13)	No (n = 86)		
Age (years)	73.77 ± 14.86	59.59 ± 16.64	0.005	
Male	4 (30.77)	49 (56.98)	0.079	2.98 (0.851–10.429)
Diabetes mellitus	3 23.08)	29 (33.72	0.450	1.69 (0.433–6.644)
Dyslipidemia	3 (23.08)	22 (25.58)	0.741	1.28 (0.308–5.281)
Type of arteriovenous anastomosis (End-to-side arteriovenous anastomosis)	10 (76.92)	76 (88.37)	0.259	2.28 (0.535–9.709)
Operation time (minutes)	80.77 ± 23.00	72.50 ± 20.00	0.189	
Site of AVF creation (Radiocephalicfistula)	9 (69.23)	70 (81.39)	0.314	1.94 (0.532–7.113)
History of AVF creation (Second time or more)	1 (0.08)	5 (5.81)	0.794	1.35 (0.145–12.567)
AVF creation before hemodialysis initiation	5 (38.46)	48 (55.81)	0.372	0.49 (0.150–1.636)

*AVF: arteriovenous fistula*

*mean ± SD for continuous variables and n (%) for categorical variables

**Student’s t test for continuous variables and Chi-squared test for categorical variables

### Risk factors for secondary AVF failure

No other risk factor for secondary AVF failure was identified other than the operation time (P = 0.038) (Table 4).

**Table 4:** Association between baseline and AVF creation characteristics and secondary AVF failure among 100 participants

Factors	Secondary AVF failure* (N = 83)		P value**	OR (95%CI)
	Yes (n = 14)	No (n = 69)		
Age (years)	67.21 ± 18.89	60.33 ± 17.14	0.182	
Male	9 (64.29)	25 (36.23)	0.051	3.168 (0.956–10.501)
Diabetes mellitus	6 (42.86)	23 (30.00)	0.348	0.667 (0.207–2.150)
Dyslipidemia	4 (28.57)	18 (26.09)	0.577	0.923 (0.246–3.469)
Type of arteriovenous anastomosis (End-to-side arteriovenous anastomosis)	11 (78.57)	59 (85.51)	0.380	1.609 (0.381–6.804)
Operation time (minutes)	84.64 ± 21.35	71.52 ± 21.18	0.038	
Site of AVF creation (Radiocephalicfistula)	11 (78.57)	54 (78.26)	0.644	0.982 (0.242–3.977)
History of AVF creation (Second time or more)	3 (21.42)	3 (4.34)	0.057	6.000 (1.071–33.605)
AVF creation before hemodialysis initiation	5 (35.71)	40 (57.97)	0.131	0.406 (0.122–1.328)

*mean ± SD for continuous variables and n (%) for categorical variables

**Student’s t test for continuous variables and Chi-squared test for categorical variables

## Discussion

It is clear that with an adequate number of qualified interventional nephrologists who can perform AVF creations, vascular surgeons’ workload and ESRD patients’ waiting times of surgical review and access placement could be reduced. Our findings including the low rates of complications and primary and secondary failures suggest that with supports from vascular surgeons, careful selection of patients and proper training, nephrologists in the low resource-settings like Vietnam can successfully perform AVF creations.

TNH is a geriatric hospital with more than 70% of patients above 60 years old [23]. Therefore, the mean age of our study participants was 61.22 ± 17.11 years old, and 62 patients were 60 years old and above. This characteristic was comparable to that of patients in similar studies conducted in the USA [24] and Korea [25], but higher than that of patients in a study conducted in India [26] which may be due to the nature of the study clinics. Regarding the etiologies of ESRD among our study participants, similar to the Indian study [26], the most common cause was hypertension, followed by diabetes mellitus. In our study, a physical examination of venous and arterial vessels in both patients’ arms was performed by the nephrologist, and difficult cases were further consulted by vascular surgeons. Due to shortage of medical imaging professionals who are familiar with Doppler vascular examination at the study clinic, only study participants with a poor vasculature identified by clinical examination underwent a preoperative Doppler ultrasound examination of both arms. This explained the low proportion of participants who had a preoperative vascular mapping with Doppler ultrasound in our study.

We found that more than half of our study participants had AVF creations before hemodialysis initiation, while only 37% of participants needed to use a temporary catheter for an urgent start of hemodialysis treatment. In contrast, a nationwide study conducted on 388 nephrologists in India to examine the current vascular access practices among nephrologists found that less than 25% of their ESRD patients started hemodialysis with AVF [27]. Similarly, in the Philippines, most CKD patients have unplanned initiations of hemodialysis treatment, and only 24% of incident hemodialysis patients start their treatment with AVF [9, 28]. Indeed, in Vietnam, a previous study conducted at the same study clinic reported that the rate of AVF creations before hemodialysis initiation was also low at 6.44% [6]. Given that nephrologists provide direct care to and can influence CKD patients, the higher rate of participants having AVF creations before hemodialysis initiation obtained in our study was probably attributable to the involvement of the nephrologist performing AVF creations who could consult participants with stage 5 CKD to undertake an AVF creation. In light of this, if nephrologists can perform AVF creations, this will help overcome delays in performing this procedure to initiate timely hemodialysis for CKD patients.

Regarding the sites of AVF creation, the radiocephalic fistula was the preferred vascular access among our participants. Our finding concurred with that of the Indian study in which, 93% of participants undertook radiocephalic fistula creations [26]. In contrast, studies in a neighboring country, Singapore, found lower rates of 58.6–67.5% of patients undertaking radiocephalic fistula creations [29, 30]. This difference was probably explained by the fact that patients in the Singaporean study had an arterial diameter of less than 2 mm, and the AVF creations were performed by vascular surgeons [29, 30]. However, another study found no statistical differences regarding the rates of radiocephalic fistula creation performed by nephrologists and vascular surgeons [2]. Indeed, the most referred site of AVF creation is the wrist (i.e., radiocephalic fistula), followed by the elbow (i.e., brachiocephalic fistula) and is selected based on the radial artery diameter [18, 31]. Interventional nephrologists usually perform AVF creations on patients having a radial artery diameter of more than 2 mm [32].

In our study, the primary AVF failure rate was 13.13% (13/99, 95%CI: 8.74–21.18%). In the Philippines, the reported primary AVF failure rate was less than 3% [9]. AVF creations are mainly performed by vascular surgeons, while less than 1% of nephrologists has been involved in interventional procedures including AVF creations [9]. This is probably attributable to the low primary AVF failure rate in this country. A study conducted in India found a comparable finding that the primary AVF failure rate associated with interventional nephrologists was 16.6% (83/500, 95%CI: 13.6–20.11%) [26]. However, our rate was lower than that of another Indian study in which the reported rate was 25.6% (90/352, 95%CI: 21.3–30.4%) due to the differences in the definitions of primary AVF failures [33]. In the latter Indian study, primary failure was defined as an inadequate AVF obtained 3 months after the operation compared to a 6-week period in our study [33]. In addition, there were reasons for the favorable outcomes of AVF creations in our study. Firstly, it could be due to our strict selection of study participants, especially a requirement of both arterial and venous diameters of more than 2 mm. Indeed, the arterial diameter is an important predictor for AVF maturation with an arterial diameter of < 2.0 mm highly associated with primary failure [33]. Secondly, the favorable outcome of AVF creations was also attributable to our considerably high rate of study participants who were well prepared for hemodialysis with AVF creations prior to the initiation of renal replacement therapy. Indeed, it is well documented that if an AVF can be established prior to the start of hemodialysis, it will create a more favorable environment with less uremia for the success of AVF surgery and fistula maturation [34, 35]. In light of our findings, to reduce the primary AVF failure rate associated with nephrologists being responsible for the AVF creation, it is pivotal to carefully select patients to have favorable conditions. It is also important to prepare patients for an AVF creation prior to the start of hemodialysis.

Regarding secondary AVF failure rate, another study reported a rate of 6.92% (31/448, 95%CI: 4.92–9.65%) with reasons including thrombosis and severe stenosis [26]. Although the secondary AVF failure in our study was also due to thrombosis and severe stenosis, the rate was higher at 16.87% (14/83, 95%CI: 10.32–26.25%). This may be explained by the fact that we did not change the needle placement sites during AVF canulation. This could also be probably due to an early cannulation for hemodialysis after 6 weeks among our study participants compared to a cannulation after 6 months as reported in another study [33]. The reason for our early use of AVF was to reduce the duration of using dialysis catheter in our patients which may lead to insufficient AVF maturation. In light of this, to prevent secondary failure of AVF created by nephrologists, AVF should be preserved for a longer period of time, probably more than 6 months as indicated elsewhere [33].

Our early complication rate was low and included bleeding and thrombosis of the anastomosis. Another similar study conducted on 216 patients in which all AVF creations were performed by a single surgeon found a higher complication rate of 22.22% and thrombosis as the most common complication [36]. Although it is unclear if the surgeon in this study was a trained nephrologist like our study, the differences in the complication rates could be due to variations in patients’ characteristics. Indeed, the mean age of their patients was 43.9 years old compared to 61.2 years old in our study [36]. We noted that these documented complications occurred in the early phase of our study and therefore, could probably be related to the limited experience of the nephrologist performing AVF creations. This could also be considered as a learning curve effect provided that such surgical technique related complications did not occur during the remaining study period. This emphasizes the importance of a strong collaboration between nephrologists and vascular surgeons in AVF creation training and managing possible complications related to surgery. At our study clinic, to become qualified nephrologists performing AVF creations, they need to complete a 6-month training on AVF creations and be an assistant surgeon of at least 60 successful AVF creations. Based on the characteristics of early surgery-related complications documented in our study, to minimize the risk of complications, we believe that a standardized AVF creation training program for nephrologists is needed. To the best of our knowledge, there is no published study or standardized guideline regarding the duration of AVF creation training for nephrologists. However, in Singapore, a local 3-year nephrology residency program and a 2-month interventional nephrology fellowship elective program are available to junior nephrologists so that they can be equipped with fundamental endovascular interventional skills [37]. We strongly believe that it is important to examine an appropriate duration of practice to attain surgical skills prior to performing AVF creations, especially in the low-resource settings like Vietnam.

The association between older age and primary AVF failure remains a controversy. A study found that age was a risk factor for primary failure [38]. Our finding concurred with this. In contrast, another study documented that older patients were less likely to have this failure [33]. It has also been documented that being female and having a second AVF creation as well as diabetes were risk factors for primary AVF failure [38–40]. A meta-analysis further found that there was an increased risk of radiocephalic fistula failure in the elderly patients and hypothesized that the use of the brachiocephalic fistulas would be better in these patients [41]. We could not find any associations between gender, diabetes, site of AVF creation and AVF failure among our patients probably due to the differences in the study contexts. Like us, some studies did not observe any association between diabetes and primary AVF failure [40, 42]. We noticed a considerably long operation time (80.77 ± 23.00 min) of 13 patients with primary AVF failure. However, there was no statistically significant association between operation time and primary AVF failure which could probably be due to the small sample size. In contrast, we found that secondary failure was associated with the operation time. Although this association has not been documented elsewhere, based on our experience, we found that the long operation time is due to patients’ small blood vessels making it difficult for AVF creations. Therefore, this increases the risk of secondary failure. In light of this, as mentioned previously, it is important to carefully select patients with an appropriate threshold diameter of blood vessels for AVF creations.

Our study has some limitations. Firstly, preoperative vascular assessment was mostly done by clinical examination with only 29% of participants undertaking vascular mapping with Doppler ultrasound. However, given the shortage of imaging professionals worldwide, preoperative vascular mapping has not been universally performed [2, 30, 43]. In our study, among 13 patients with primary AVF failure, seven underwent preoperative vascular mapping. Among 14 patients with secondary AVF failure, five underwent this imaging test. Our findings suggest that an adequate clinical examination by qualified health professionals could probably be an efficient alternative to the preoperative vascular mapping in settings where the availability of this imaging test is limited, although more robust studies are needed. Similarly, patients with AVF creation failures were assessed based on clinical examination. Without Doppler ultrasound, stenosis leading to AVF failures may be misdiagnosed as thrombosis. Secondly, the impact of mineral metabolism disturbances on AVF maturation remains controversial [44, 45]. Despite this, the potential association between the measurable markers of mineral metabolism and functional AVF maturation was not examined in this study. Thirdly, given that AVF creation performed by a trained nephrologist is comparatively a new approach in developing countries, our sample size was small. Therefore, future studies with a larger sample size will be better representative of the general population. Fourthly, our 18-week follow-up period was considerably short. AVF failures may occur after the follow-up period and thus, could not be recorded. We believe conducting similar studies with a longer follow-up is essential to have a full understanding of this issue.

## Conclusions

Our findings confirm that with supports from vascular surgeons, careful selection of patients and proper training, nephrologists can successfully perform AVF creations. It is important to follow up patients with old age and those with a long operation time to detect primary failure and secondary failure, respectively. To increase successful rates, it is important to have a standardized AVF creation training program and practice for nephrologists. Future studies with larger sample sizes and longer follow-ups are needed to provide more robust evidence on the role of nephrologists in performing AVF creations.

## Data Availability

The datasets used and/or analyzed during the current study are available from the corresponding author on reasonable request.

## Declarations

### Informe concent

All methods were carried out in accordance with the Declaration of Helsinki.

## List of abbreviations

AVF: Arteriovenous fistula
AVG: Arteriovenous graft
CKD: Chronic kidney disease
CAPD: Continuous ambulatory peritoneal dialysis
ESRD: End-stage renal disease
ETS: End-to-side
STS: Side-to-side
TNH: Thong Nhat Hospital
